# COVID-19 outbreak control, example of ministry of health of Turkey

**DOI:** 10.3906/sag-2004-187

**Published:** 2020-04-21

**Authors:** Yasemin DEMİRBİLEK, Gülen PEHLİVANTÜRK, Zeynep Özge ÖZGÜLER, Emine ALP MEŞE

**Affiliations:** 1 General Directorate of Public Health, Ministry of Health, Ankara Turkey; 2 Ministry of Health Ankara Turkey

**Keywords:** COVID-19, COVID-19 pandemic, control, preventive measures

## Abstract

Our first COVID-19 case in Turkey was a 44-year-old male who referred to the hospital on March 9, 2020. The first related death occurred on March 17, 2020. Preparedness for the pandemic has been ongoing before the first case was detected. The National Pandemic Plan was published in 2006. The Pandemic Influenza National Preparedness Plan was available after being updated in light of experiences gained during the 2009 Influenza A pandemic. Accordingly, Pandemic Coordination Boards and Operation Centers have been established on the national and provincial levels. This was an adaptable plan to the Novel Coronavirus Disease (COVID-19). We formed teams to work on a 24/7 basis and established a Scientific Committee at the Public Health Emergency Operation Center within the General Directorate of Public Health. “COVID-19 Risk Assessment”, “COVID-19 Guideline” and “Case Report Form”, regulations of personal protective equipment along with need-based guidelines, treatment algorithms, brochures and related documents have been released. For the case-based follow-up, Public Health Management System (HSYS) is being used. PCR and rapid diagnostic kits are being used to analyze the samples at the central Microbiology Reference Laboratory and the authorized laboratories in several provinces. Various preventive measures were implemented including flight restrictions to certain countries, gradually expanded to suspending all flights and prohibiting the entry of foreign nationals, 14-day isolation and symptom monitoring for those that came from countries under risk. Persons with chronic diseases have been granted an administrative leave, on campus education at schools and activities of public rest and entertainment areas were temporarily suspended. The measures have been implemented for penitentiary institutions, dormitories, nursing homes, public transport and intercity buses, and also weekend curfews are implemented. In accordance with the pandemic plan, actions have been carried out with a multi-sectoral approach, and preventive measures have been implemented to cover the society as a whole.

## 1. Introduction

Epidemic diseases that spread widely over multiple countries or continents around the world are called pandemics [1].

The National Pandemic Plan was published in 2006 as a part of the preparation for influenza pandemic in our country [2]. The plan was molded into its final form as: “The Pandemic Influenza National Preparedness Plan” after being updated in the light of experiences gained during the 2009 Influenza A pandemic along with the regulations and recommendations made by WHO during the process. The Pandemic Influenza National Preparedness Plan was prepared under the coordination of the Ministry of Health, General Directorate of Public Health (GDPH) in collaboration with other institutions and organizations. The Plan was published in the Official Gazette as the Presidency Circular 2019/5.

The Pandemic Influenza National Preparedness Plan has been prepared to provide an outline of the minimum elements needed to be prepared, as well as to ensure optimal readiness. The plan aims to secure the continuity of public services and to reduce the transmission of the pandemic strain, number of patients related to the pandemic, hospitalization and deaths due to the disease, and the socioeconomic burden formed by the pandemic.

Provinces were requested to generate “Provincial Pandemic Influenza Preparedness and Action Plans” in line with the Pandemic Influenza National Preparedness Plan. In compliance with this request, 81 Provincial Health Directorates prepared drafts of “Provincial Pandemic Influenza Preparedness and Action Plans”. The committee evaluated these plans, and provinces were asked to complete their preparations in accordance with the feedbacks given on a provincial basis.

Even though the Pandemic Influenza National Preparedness Plan has been prepared for Pandemic Influenza, this plan is adaptable to the New Corona Virus Disease (COVID-19) caused by a virus that transmits via respiratory droplets, similar to Influenza [3,4].

In December 2019, cases of pneumonia of unknown etiology in Wuhan, China, Hubei Province were reported. On January 9, 2020, the first COVID-19-related death reported from China. On January 17, 2020, the first imported cases were reported from Thailand and Japan. Towards the end of January, cases started to appear in Europe. The World Health Organization (WHO) declared a pandemic on March 11, 2020. On March 14 2020, Europe was declared as the epicenter of the epidemic by WHO [5,6].

## 2. Implemented measures and policy responses to COVID-19

Since it was reported by WHO on January 5, 2020 that 59 suspicious cases were detected between December 31, 2019–January 5, 2020 in the People’s Republic of China the disease has been followed up by GDPH in our country. We have been closely monitoring the developments in the world and the international spread of the disease.

We formed teams to work on a 24/7 basis, and we established a Scientific Board for COVID-19 at the Public Health Emergency Operation Center within the GDPH. We have taken necessary measures in our country simultaneously with the recommendations of WHO [5,7].

Scientific Board for COVID-19 conducted the “COVID-19 Risk Assessment” on January 22, 2020. In addition, “COVID-19 Guideline and Case Report Form” was prepared in the same meeting. The ‘’ COVID-19 Disease Guideline’’ includes general information about the infection, case definitions and information on case management, infection control and isolation, patient care and treatment. The guideline also included information for the people who will be travelling to the countries with COVID-19 cases. This guidance has provided a standardized approach all over the country towards suspected cases. The first version of the guideline was published on January 24, 2020. Following the developments, we constantly update it and publish it on the website of the Ministry of Health [8]. 

The Scientific Committee, staying up-to-date on international developments, continues to meet at a minimum of two times a week to present opinions and suggestions to the Ministry of Health in the light of scientific developments. Answers to “Frequently Asked Questions”, need-based guidelines, presentations, treatment algorithms, posters, brochures and all related documents are being updated. New documents are prepared and shared in accordance with the current scientific developments, course of the disease in our country and experiences. The reports, visual materials, answers to frequently asked questions for both healthcare professionals and the public are shared on the aforementioned website [8].

In our country, the first sample was analyzed at the GDPH Microbiology Reference Laboratories after being taken on January 21, 2020. Currently, we take samples of the persons that meet the current possible case definition continuously. Results of the samples tested using polymerase chain reaction (PCR) are shared in a timely manner. In order to ensure rapid operation, the respiratory samples are being analyzed not only at the central laboratory but also at the laboratories that are authorized by the central GDPH Microbiology Reference Laboratory in several provinces. In addition, rapid diagnostic kits are being used. We provide all the materials required for sampling and diagnosing, at the central level and distribute them to the provinces.

Training about the COVID-19 was provided both to healthcare personnel and to the public. To disseminate information, different communication channels such as radio, television and social media are being used in order to reach the whole public. Public educative materials include information on the disease, ways to prevent the disease and general hygiene rules. People travelling abroad have been requested not to leave their homes and not to accept guests for 14 days, even when there are no signs of the illness. If they have to leave the house, they are advised to use masks; and in case of developing symptoms of infection such as fever, cough, and sore throat they are recommended to refer to the nearest health institution while using masks. It has been emphasized that people should avoid entering crowded environments, people with signs of infection such as cough, runny nose, sneezing, should use masks, and people should close their mouths with disposable wipes when they cough and sneeze. Importance of frequent airing of indoor environments, washing hands frequently with soap and water, paying attention to general cleaning rules and social distancing to protect against infection is also stated.

In line with the Pandemic Influenza National Preparedness Plan, Pandemic Coordination Boards and Operation Centers have been established on the national and provincial levels. The National Coordination Board held its first meeting on March 10, 2020. The first case in our country was detected on the same day. Our first case was a 44-year-old male, whose symptoms started on March 03, 2020 and who referred to the hospital on March 9, 2020. The first death case related to COVID-19 occurred on March 17, 2020, when the total number of cases had reached 98.

Preparations were made for personal protective equipment (PPE) and medications, and they were distributed. Additionally, following the current developments, drugs suitable for use have been identified, supplied and distributed to all patients with an indication that is determined by the health institutions. Updates of necessary PPE, medication and other material is ongoing. There are no current problems in our country in terms of the supply of medication and materials.

Individuals who meet the current definition of the possible case described in the COVID-19 guideline are being monitored until their results are available. For the case-based follow-up, Public Health Management System (HSYS) is being used. With the case tracking module created under the HSYS program, all the COVID-19 possible cases (starting from their detection), people who come from abroad and who need isolation at home and the contacts of the COVID-19 cases are monitored including their hospitalization process. As of March 17, 2020, the data has been entered retrospectively to enable the access of the old data through the module.

In addition, the close contacts of cases, people in the risk groups and patients who are being followed at home within the HSYS module are also being followed by field teams and family medicine units.

Symptom monitoring and 14-day isolation were performed for those that came from countries under risk and from Umrah. Possible case management algorithms were applied if symptoms occurred in these individuals.

Our citizens and their families have been discharged from the quarantined areas, such as Wuhan and from the areas with high numbers of case. On their arrival to our country, health checks were made, samples were taken, and they were followed up for 14 days.

Data related to the disease are shared daily inside our country and with the world [8]. Within the scope of the International Health Regulations (IHR), international and domestic flights, close contacts of cases are monitored and notified to the relevant countries [9].

Specific information about the disease, on issues to be considered and infection control measures were given to relevant institutions and organizations. Working in cooperation with other ministries, institutions and organizations, various suggestions have been made regarding pandemics. In line with these suggestions, relevant institutions conducted studies regarding necessary control measures, decisions were taken and implemented accordingly.

Nonemergency surgeries and nonurgent dental practices have been postponed in order to ensure preventing disease transmission and to keep healthcare institutions sufficient in capacity. In each province, pandemic hospitals have been determined. Possible and confirmed cases are admitted and treated in isolation in the Pandemic Hospitals (Ministry of Health hospitals, State and Foundation University hospitals and private hospitals).

In order to prevent the disease from entering and spreading in our country, all public officials travel abroad permissions have been stopped as of 12 March 2020.

The entry of foreign nationals to our country is temporarily prohibited, while Turkish citizens and people who have a residence permit in Turkey are allowed to enter. At the entrance of our country, people were questioned in terms of symptoms; those without symptoms were isolated and followed at their homes for 14 days. The people who were brought to our country collectively with the planned flights from abroad and had no symptoms were taken to the designated dormitories for 14 days and were followed up. Public officials were assigned to have administrative leave during the period of isolation, and a report was issued for other employees. Those with symptoms were managed in health institutions in accordance with the case management algorithms.

Follow-up of the cases that were admitted to health institutions or monitoring of the persons who were close contacts of cases were terminated according to the discharge and isolation rules and algorithms [10].

Measures have been taken for all flights coming from areas under risk. Announcements were made to the passengers at planes. Passengers entering our country were scanned with thermal cameras; passengers whose fever was detected were evaluated by the Health Inspection Center and managed according to the current case management algorithm [10]. Health control measures have been implemented at land, air, sea entry points and ships.

Since the beginning of February 2019, restrictions on travel have been applied to regions with a high number of cases, and the scope of the travel restriction has been gradually expanded in compliance with the spread of the disease. Restrictions have been introduced for people coming to our country by land border gates, sea border gates and airways. The people who have been in the areas under risk in the last two weeks and who do not have a residence permit in our country were not allowed to enter the country. The people allowed to enter our country were evaluated in terms of disease symptoms, those with symptoms were isolated in health institutions and samples were taken. Those without symptoms were followed at home for 14 days. There is no restriction for the citizens of other countries to leave our country.

The first travel restrictions have been applied to the people coming from the People’s Republic of China during the first half of February and to Iran as of February 23, 2020. For those who came to our country before the implementation of travel restrictions were followed up for 14 days in designated hospitals near the border if they had a history of being in the regions with a high number of cases, such as the city of Kum, or had contact with or visited a COVID-19 patient.

The application of ceasing flights and measures implemented at the borders for people who had been in China and Iran have been expanded to include South Korea, Italy and Iraq on March 3, 2020; Germany, Spain, France, Austria, Norway, Denmark, Sweden, Belgium and the Netherlands on March 14, 2020. On March 15, 2020, Sarp, one of the three border gates between Turkey and Georgia, was mutually closed to passenger traffic. Reciprocal travels with Azerbaijan, air transport, land and airport border gates were closed as of March 17, 2020. Two other border gates between our country and Georgia were closed to passenger traffic on March 18, 2020. Precaution measures for passenger entrances were initiated at all border gates on March 17, 2020, for Saudi Arabia, the United Arab Emirates, Ireland, Switzerland, the UK and Egypt. The implementation of restrictive measures for the border crossings for Greece and Bulgaria started on March 18, 2020. On March 21, 2020 precautions were taken at the border crossings, and the flights have been stopped for several other countries including Kuwait, Bangladesh, Mongolia, Turkish Republic of Northern Cyprus, Ukraine, Kosovo, Morocco, Lebanon, Jordan, Kazakhstan, Uzbekistan, Oman, Slovenia, Moldova, Djibouti, Equatorial Guinea, Canada, India, Hungary, Guatemala, Poland, Kenya, Sudan, Chad, Philippines, Latvia, Taiwan, Peru, Sri Lanka, Ecuador, Niger, Tunisia, Algeria, Ivory Coast, Finland, Angola, Czechia, Dominican, Cameroon, Montenegro, Colombia, The states of North Macedonia, Mauritania, Nepal, Portugal and Panama. After March 27, 2020, all international flights were cancelled.

Advice on the trade was given in regards to the disease. Freight and cargo transportation continued in line with the determined procedures. COVID-19 related prevention and control measures were implemented for airport personnel, airline companies, their flight crew, baggage and cargo personnel and cargo flights that fly to the regions where the case numbers are high. 

Suggestions for workplaces and offices aimed to prevent the spread of the disease were taken and implemented. Measures including flexible working time tables and working home-office to decrease the close contacts of the employees were applied. Pregnant women, mothers who have legal milk leave, disabled employees, people over 60 years old (except those in executive positions), disadvantaged groups (those with immune problems, cancer, chronic respiratory diseases, obesity and diabetes, cardiovascular diseases and organ transplant patients, and overall chronic diseases patients) have been granted an administrative leave starting of March 16, 2020.

Measures have been taken for common areas such as dormitories, nursing homes, military barracks and similar public living areas, accommodation facilities, hotels and restaurants. Basic infection protection and control principles are decided on and implemented covering hygiene rules, actions to be taken in the presence of people with symptoms associated with the disease at workplaces and suggestions for the work environment and workplace contacts of positive cases.

Information notes were sent to schools and parents. Education has been suspended at schools and universities as of March 16, 2020 and distance education is still ongoing.

Measures for disease protection and control have been taken for indoor spaces open to public usage such as airports, bus stations, train stations, shopping malls, cinemas, theaters, etc., and hygiene and cleaning advices have been applied. To minimize the risk of COVID-19 transmission in the future; restrictions have been implemented for the areas where people can gather (entertainment venues, theater, wedding hall, mosque, tea garden, local etc.) or where there is a risk of infection (hairdresser, barber, beauty salon etc.). The risk of transmission of the disease is high for places that operate as Public Rest and Entertainment Areas and where citizens can be in contact at a very close distance. For this reason, pavilion, discotheque, bar, night club, theater, cinema, show center, concert hall, engagement/wedding hall, musical/music restaurant/cafe, casino, pub, tavern, coffeehouse, café, cafeteria, country garden, hookah hall, hookah cafe, internet lounge, internet cafe, all kinds of game halls (arcade, playstation etc.), all kinds of indoor playgrounds (including shopping malls and restaurants), tea garden, association lounges, amusement park, swimming pool, Turkish bath, sauna, thermal pool, massage parlor, SPA, sports centers and condolence houses’ activities were temporarily suspended as of March 16, 2020. Besides, all meetings and events of civil society organizations (associations, foundations) and organizations that bring people together, including training, were suspended as of the same date.

All kinds of scientific, cultural, artistic and similar meetings or activities to be held in open and closed areas at national and international level have been postponed as of March 20, 2020.

As of March 21, 2020, seating areas in all restaurants, patisseries and similar workplaces were removed and only takeaway service is allowed. On the same date, the activities of barbershops, beauty salons/centers, hairdressers etc. were suspended.

In order to prevent the spread of the disease in penitentiary institutions, measures have been implemented for the residents, and personnel hygiene measures have been increased. As of the second week of March, no admissions have been made without passing the health check. Activities that may increase the risk of disease transmission, such as visits, special leave rights, hearings, activities carried out by people living in different wards and transfers have been postponed. Actions to take in case of detecting a person with the symptoms are decided on. Measures have been implemented to ensure that preventive and therapeutic health services are carried out in the best way. The convicts/detainees to be released were informed on the disease and its prevention and leaflets were distributed.

In order to stop the spread of diseases in public, a restriction has been implemented in the form of a curfew to those who are over the age of 65 on March 21, 2020, those with chronic disease, and those under the age of 20 on April 3, 2020. Vefa Social Support Group was created to meet the needs of those who have to stay at their homes. Travel Permits are issued onnecessarysituations.

In order to reduce human travelling and associated disease transmission, road and air travels are allowed to be done after obtaining a permit. As of March 28, 2020 intercity buses at the city entrances and exits for our 30 metropolitan cities (as of April 3, 2020, Zonguldak province was included) have been restricted. Starting on April 11, 2020, curfews and quarantine measures are implemented on weekends for the same provinces, with the exception for certain sectors.

Measures for disease prevention and control are implemented, and hygiene and cleaning advices are applied for the transportation vehicles such as public transportations and shuttle buses. As of March 21, 2020, restrictions have been imposed on the number of passengers (50% of the capacity) in public transport and of working hours and allowed customer numbers in the markets.

It is compulsory to use a mask in environments such as markets, marketplaces and buses where there is a possibility that social distance cannot be achieved. Apart from food and cleaning materials, the sale of other products such as clothing and toys has been stopped in the marketplaces and measures have been taken to increase the personal distance.

In coordination with the Directorate of Religious Affairs, the public was informed on Friday, March 06, 2020, before the case was detected in our country. After March 16, 2020, performing prayers with the congregation, including Friday prayers are suspended.

Disease prevention and control measures are taken for the people working in agricultural production, especially seasonal workers, and they were informed on the disease.

The morgue and burial procedures for the people who died due to the disease and the autopsy procedures to be carried out in probable cases are defined. Moreover, the personal protective equipment to be used is specified for workers of those fields.

Irregular migrants who have been found/evaluated to have entered our country after being in countries (the last 14 days) where the disease is intensely identified, first receive health examinations. Those with disease symptoms are evaluated in health institutions according to case algorithms, and their samples are taken in the hospital. Those with no symptoms are isolated for 14 days in temporary shelter camps or in centers located in provinces to be taken under observation. The physicians assigned by the relevant Provincial Health Directorate follow them up for symptoms every day, and those who develop symptoms during this period are evaluated in health institutions according to the case algorithms. At the end of this period, procedures and actions determined by the Directorate General of Migration Management are carried out for those who have no symptoms within the framework.

In Migrant Health Centers, migrants are trained on the disease, and its prevention and control measures. Materials such as posters and brochures prepared on the subject are translated into Arabic, English and Persian languages ​​in order to ensure that people have access to correct information.

Provided by the Ministry of Health for disease protection: everyone regardless of whether they have social security, can utilize personal protective equipment, diagnostic tests and medications used for related treatment. Actions are carried out to supply masks to the public free of charge.

Detailed studies are carried out on various kinds of subjects such as diagnosis, treatment, prevention, medical waste management for the pandemic.

Scientific studies are conducted on subjects such as virus isolation, vaccine, drug and plasma treatment. Real-time PCR diagnostic kit for detecting SARS-CoV-2 in respiratory tract samples has been developed by the Ministry of Health GDPH Microbiology Reference Laboratories and Biological Products Department Virology Laboratory. Thus, there was no limitation in the number of diagnostic tests.

## 3. Conclusion

Although being well prepared, the pandemic is a process that needs fast actions and brings out challenges for all countries. The challenges experienced in our country are not more than those experienced by many countries in the world. We initiated monitorization of the disease in the early period; accordingly, measures were taken early, and the entrance of the disease in our country was delayed. Most of the preparations were completed during this period. Having sufficient hospital beds and intensive care unit capacities for nearly all of our provinces was evaluated as an advantage. In addition, personal protective equipment such as gloves, medical and respiratory masks, gowns, goggles/face protectors are produced in our country. Thus, shortness of PPE has not occurred, and aid has been provided to several countries.

Measures have been taken with caution to ensure that flight restrictions and the measures taken at border crossings do not affect international relations.

Regarding the pandemic, the developments around the world and in our country were monitored continously, and updates regarding the applications were implemented and announced rapidly. The pandemic has been responded according to the most suitable measures for the situation in our country. First, precautions were taken to prevent the disease from entering the country. After the disease was seen in our country, actions were carried out to prevent and treat the spread of the disease. In accordance with the pandemic plan, actions have been carried out with a multi-sectoral approach, and preventive measures have been implemented to cover the society as a whole.
